# Comparative impacts of normobaric vs. hypobaric hypoxia on tissue integrity and gut microbiota in acute high-altitude murine models

**DOI:** 10.1128/spectrum.02214-25

**Published:** 2026-02-13

**Authors:** Xiaohong Liu, Yameng Zheng, Xiaohong Tian, Jiaojiao Ma, Yuanzhe Li, Chengcheng Zhao, Chenxu Zhang, Juan Liu, Hexiang Huang, Yingchao Wang, Chi Tang, Kangning Xie, MingMing Zhai

**Affiliations:** 1 The College of Life Sciences, Northwest University 628998 , Xi’an, Shaanxi, China; 2 School of Biomedical Engineering, Fourth Military Medical University 12644, Xi’an, Shaanxi, China; 3 Shaanxi Provincial Key Laboratory of Bioelectromagnetic Detection and Intelligent Perception, Xi’an, Shaanxi, China; Nova Southeastern University, Fort Lauderdale, Florida, USA

**Keywords:** gut microbiota, hypobaric hypoxia, normobaric hypoxia, 16S rRNA

## Abstract

Normobaric hypoxia (NH) and hypobaric hypoxia (HH) are the two primary approaches currently used to establish high-altitude animal models in lowland laboratories. However, their relative suitability and effectiveness remain debated. This study conducts a comparative assessment of acute high-altitude models generated under NH and HH, specifically comparing their impacts on gut microbiota. Mice from the same batch were exposed to NH or HH conditions equivalent to 5,500 m in a high-altitude simulation chamber. After 72 h of exposure, tissue samples were collected. Histopathological analyses showed that HH caused more severe tissue injury than NH across multiple organs, particularly the lung, liver, hepatic lobule, and prefrontal cortex. Additionally, 16S rRNA sequencing characterized changes in gut microbiota structure, composition, differentially abundant taxa, and predicted functional profiles. These analyses revealed distinct gut microbiota structure between the two hypoxic conditions. The HH group showed a greater increase in Bacillota and a more pronounced decrease in Bacteroidota, along with a higher abundance of *Lactobacillus*. This comparative evaluation of NH and HH not only provides a critical reference for model selection in high-altitude gut microbiota research but also lays the groundwork for establishing more reliable and optimized experimental modeling strategies for future high-altitude studies.

**IMPORTANCE:**

This study demonstrates that hypobaric hypoxia induces more severe tissue injury and more pronounced shifts in gut microbiota composition than normobaric hypoxia at 5,500 m. These results inform experimental design choices and emphasize the need for careful model selection to improve data reliability and strengthen the connection between theoretical research and modeling in high-altitude physiology.

## INTRODUCTION

At high elevations, barometric pressure (PB) declines with increasing altitude, which reduces the inspired fraction of oxygen (F_I_O_2_) (1). Specific combinations of PB and F_I_O_2_ determine the inspired partial pressure of oxygen (P_I_O_2_) (2). It has long been assumed that decreases in P_I_O_2_ directly contribute to major high-altitude disorders, including acute mountain sickness (AMS) and high-altitude cerebral edema. According to this assumption, identical P_I_O_2_ values resulting from different PB and F_I_O_2_ conditions should elicit comparable physiological responses. For this reason, normobaric hypoxia (NH) and hypobaric hypoxia (HH) are commonly used in low-altitude laboratories to simulate high-altitude P_I_O_2_ when studying AMS (3). Some studies report that submaximal cycling exercise during acute NH exposure produces cardiopulmonary responses similar to those observed under HH (4–7). However, the equivalence of HH and NH remains contested. Multiple investigations show that both the prevalence and severity of AMS are higher under HH than NH (8–11). These observations suggest that NH, although capable of generating the same P_I_O_2_ as HH, may lead to distinct physiological outcomes.

High-altitude (plateau) hypoxia induces systemic hypoxia that disrupts the intestinal barrier and perturbs the gut microbiota (12, 13). Increasing evidence demonstrates that gut microbiota are essential for mammalian health (14). Their metabolites interact with host organs, tissues, and cells and contribute to defense against pathogen colonization (15). Under normal conditions, the gut microbiota and host coexist in a dynamic, mutually beneficial relationship, but exposure to plateau hypoxia alters this equilibrium (12, 13). For example, mountaineering expeditions at high altitudes decreased beneficial bifidobacteria and increased potentially pathogenic gram-negative bacteria, particularly certain Enterobacteriaceae, such as *Escherichia coli* (16). Other studies report shifts in microbial enzyme activities, with increases in protease and polyphenol hydrolase and decreases in phosphatase (17). Collectively, these findings indicate that both composition and function of the intestinal microbiota change under hypoxic conditions (12).

A few studies have characterized how HH affects gut microbiota composition, reporting increased bacterial translocation and compositional shifts following HH exposure (18–20). To the best of our knowledge, no study has compared gut microbiota responses between NH and HH at the same P_I_O_2_. Therefore, clarifying whether the two modeling approaches produce consistent effects on the microbiota is necessary. To address this, we simulated an altitude of 5,500 m under either NH or HH for 3 days. We compared organ pathology by hematoxylin and eosin (H&E) staining and assessed microbiota diversity, composition, and key taxa using full-length 16S rRNA gene sequencing on the PacBio SMRT platform to determine whether the two hypoxia models yield equivalent gut-microbiota outcomes.

## MATERIALS AND METHODS

### Animals

C57BL/6 male mice (7 weeks old; 14 ± 1 g) were obtained from the Lab Animal Center of the Fourth Military Medical University. Animals were maintained under specific pathogen-free conditions in a climate-controlled room (23 ± 1°C; 45 ± 5% humidity) with a 12/12 h light–dark cycle and provided *ad libitum* access to a standard laboratory diet and water. A total of 24 mice were randomly assigned to cages and then allocated to three experimental groups to minimize cage-related effects.

### Acute hypoxia modeling

The normobaric normoxia (NN) group served as the control and was maintained at an altitude of 500 m, corresponding to a local atmospheric pressure of 95.2 kPa and 20.9% O_2_. NH and HH exposures were generated using specialized environmental chambers (TOW-Int Tech, Shanghai, China). According to the equivalent air altitude (EAA) model formula $P_{I}O_{2}=\left(P_{B}-47\right)\times F_{I}O_{2}$ (the partial pressure of water vapor at 37°C is 47 mmHg) (21), the $P_{I}O_{2}$ of 5,500 m is 9.35 kPa. The NH group was therefore exposed to 95.2 kPa with 10.5% O_2_, while the HH group was exposed to 51 kPa with 20.9% O_2_. These conditions ensured that the EAA of the NH and HH groups remained matched throughout the experiment.

Both chambers provided real-time monitoring and precise control of environmental parameters. The ProOx-810 hypobaric chamber maintained an altitude control accuracy of ±100 m, temperature at 23 ± 2°C, and humidity at 53 ± 2% RH. The ProOx-100 normobaric chamber ensured gas control precision of O_2_ ± 0.1% and CO_2_ ± 0.1%, temperature at 23 ± 2°C, and humidity at 53 ± 2% RH. All parameters were logged in real-time to ensure stability throughout the experiments.

### Histological analysis

At the designated time points, mice were deeply anesthetized by intraperitoneal injection of sodium pentobarbital (100 mg/kg body weight). The absence of a pedal reflex confirmed an adequate surgical plane of anesthesia. Cervical dislocation was then performed as a secondary physical method to ensure death. The abdominal cavity was opened, and the target tissues were excised without delay. Tissue samples were immediately immersed in 4% paraformaldehyde for fixation. After paraffin embedding, consecutive sections were prepared, stained with H&E, and digitally scanned (22). The slides were examined using CaseViewer 2.2 for histological evaluation. Tissue lesion severity was assessed using a five-point scale in which 0 indicates no or minimal lesions, 1 indicates mild lesions, 2 indicates moderate lesions, 3 indicates severe lesions, and 4 indicates extremely severe and extensive lesions.

### 16S rRNA PacBio SMRT gene full-length sequencing

Three to five grains of fresh fecal pellets were collected in sterile tubes and stored at −80°C for 16S rRNA PacBio SMRT full-length sequencing. Total microbial genomic DNA of fecal samples was extracted using the EZNA feces DNA kit (Omega, GA, USA) according to the manufacturer’s instructions. PCR amplification of full-length bacterial 16S rRNA genes employed the forward primer 27F (5′-AGAGTTTGATCMTGGCTCAG-3′) and reverse primer 1492R (5′-ACCTTGTTACGACTT-3′) as previously described (23). Each PCR reaction (25 μL) contained 12.5 μL of 2× KAPA HiFi HotStart ReadyMix, 3 μL of barcoded forward primer (2.5 μM), 3 μL of barcoded reverse primer (2.5 μM), 5 μL of template DNA (approximately 1–2 ng total), and nuclease-free water to volume. PCR was performed under the following cycling conditions: initial denaturation at 95°C for 3 min; 25 cycles of 95°C for 30 s, 57°C for 30 s, and 72°C for 60 s; followed by a final extension at 72°C for 5 min. Amplicons (~1.5 kb) were verified by agarose gel electrophoresis prior to equimolar pooling and SMRTbell library construction.

### Bioinformatics analysis

Bioinformatics analyses were performed to evaluate amplicon sequence variants (ASVs), their relative abundances, sample diversity, principal component patterns, and inter-sample differences.

### Sequencing data processing

The multi-round sequencing data for each DNA template were merged into high-accuracy CCS reads, after which Lima v1.11.0 was used to split CCS reads from different samples according to barcode sequence. Reads lacking both primers or falling outside the 1.2–1.8 kb length range were removed. Chimeric sequences were identified and filtered using DADA2 to generate high-quality CCS reads for downstream analyses (Table S1).

### Species annotation and taxonomic analysis

Using QIIME v2021.4, ASV sequences were aligned to the Greengenes reference database to obtain corresponding taxonomic classifications. Community composition for each sample was then quantified at multiple taxonomic levels (phylum, class, order, family, genus, and species), and species abundances were calculated accordingly. To ensure equitable comparison of microbial diversity across samples, all sequences were rarefied to an even depth of 5,258 sequences per sample for both alpha and beta diversity analyses. Beta diversity analyses in QIIME were used to assess similarity in species composition across samples.

### Statistical analysis

Statistical analyses were performed using GraphPad Prism 9.4. Alpha diversity indices (Ace, Chao1, Shannon, and Simpson) were compared using Wilcoxon rank-sum tests for two-group comparisons and Kruskal–Wallis tests for multi-group comparisons. The Kolmogorov-Smirnov test was applied to assess normality, and the Levene test was used to examine homogeneity of variance. Group differences were evaluated using one-way ANOVA followed by Bonferroni’s post hoc tests for multiple comparisons. Data are reported as mean ± SEM, and statistical significance was defined as *P* < 0.05.

## RESULTS

### Observation of histological injury

**Fig 1:**
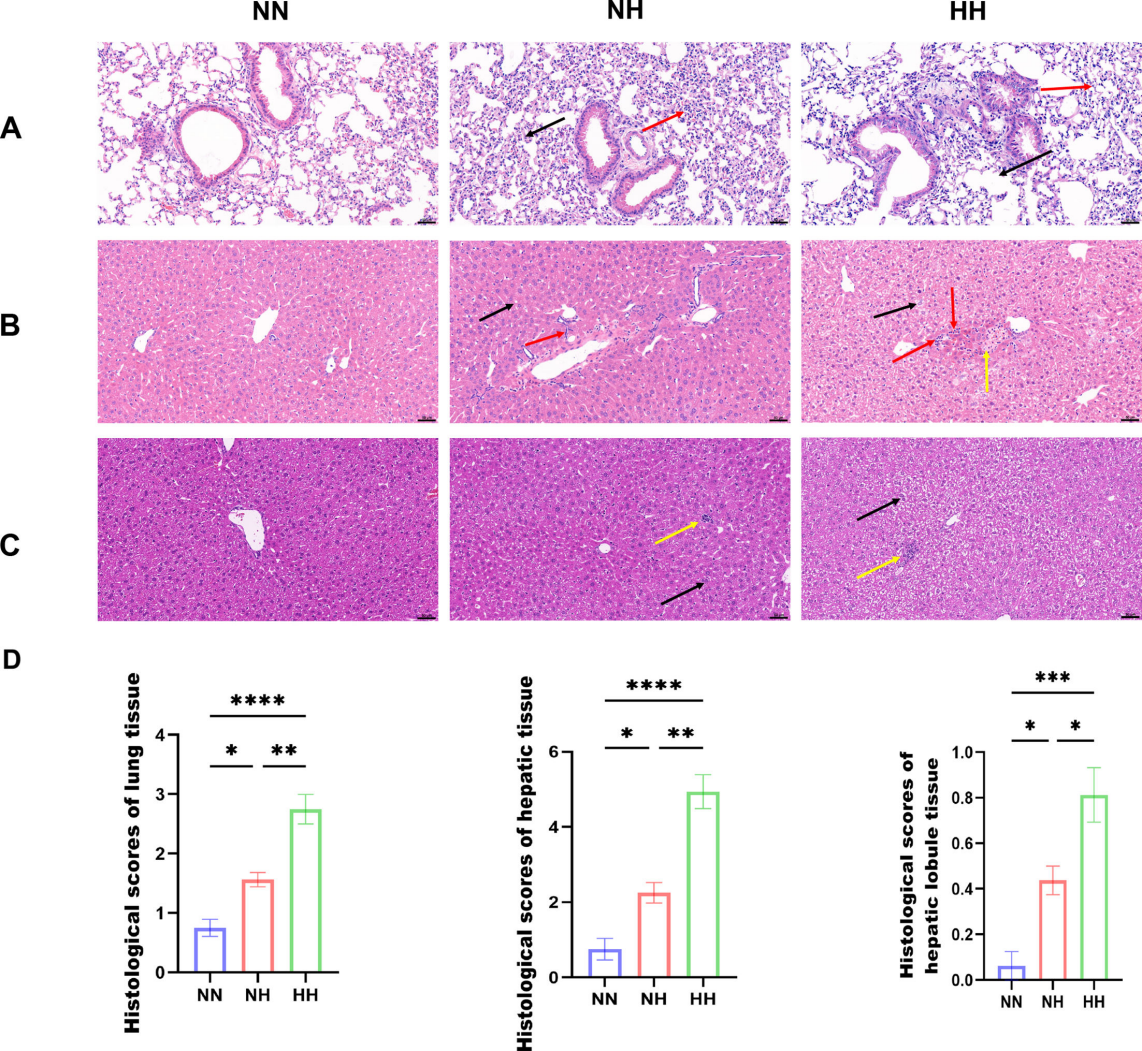
Histological injury in different groups of mice. (**A**) Representative images of H&E staining of lung tissues. (**B**) Representative images of H&E staining of hepatic tissues. (**C**) Representative images of H&E staining of hepatic lobule tissues. Scale bar: 50 μm. (**D**) Histological scores. All data are presented as the mean ± SEM. (*n* = 8; *P < 0.05, **P < 0.01, ***P < 0.001, ****P < 0.0001).

H&E staining revealed that the tracheal epithelium of the NN group remained intact, with normal cell morphology, tightly arranged structures, clear alveoli, and no appreciable alveolar wall thickening. In the NH group, alveolar walls were thickened in several regions (red arrows), accompanied by scattered granulocyte infiltration (black arrows). In the HH group, alveolar wall thickening was minimal. However, mild granulocyte infiltration (red arrows) and pronounced alveolar dilation were present, compressing adjacent alveoli and narrowing the surrounding airspaces (black arrows) (Fig. 1A). In liver tissue, the NN group displayed well-defined hepatic lobules, orderly sinusoids, and no evidence of inflammation. In the NH group, hepatocytes contained variably sized cytoplasmic vacuoles (black arrows), and localized bile duct proliferation was observed (red arrows), although inflammation was absent. In the HH group, hepatocyte edema was prominent, characterized by loosely arranged, lightly stained cytoplasm (black arrows). Localized hepatocyte necrosis was also detected, marked by nuclear fragmentation or dissolution (yellow arrows), accompanied by limited lymphocyte and granulocyte infiltration (red arrows) (Fig. 1B). The NN group again exhibited distinct hepatic lobules, with orderly arranged sinusoids and no significant signs of inflammatory cell infiltration. The NH group maintained clear hepatic lobular architecture but showed widespread mild vacuolar degeneration, with cytoplasmic swelling and irregular vacuoles (black arrows), and occasional localized inflammatory foci (yellow arrows). In the HH group, hepatic lobules remained well organized, yet moderate vacuolar degeneration affected a large proportion of hepatocytes, particularly near the bile ducts. This degeneration, characterized by cytoplasmic swelling and variably sized vacuoles (black arrows), was accompanied by occasional inflammatory foci (yellow arrows) (Fig. 1C).

**Fig 2:**
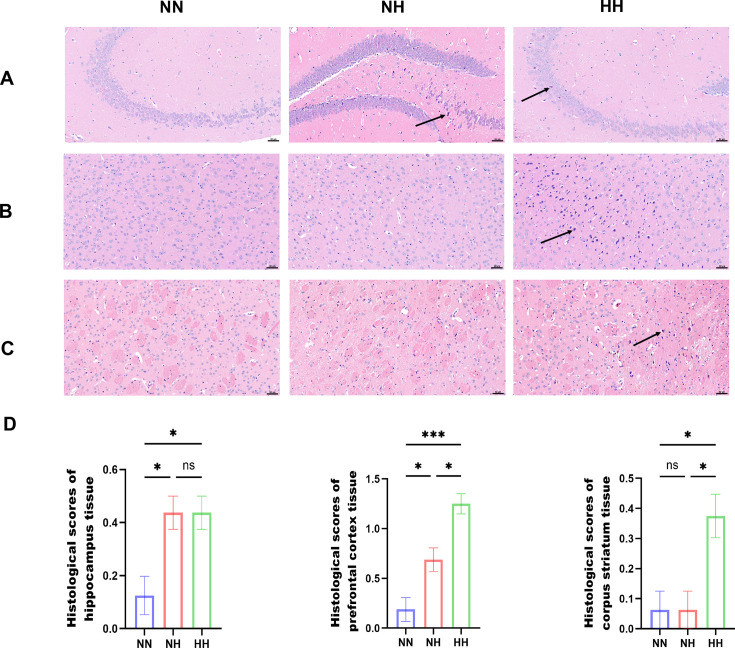
Histological injury in different groups of mice. (**A**) Representative images of H&E staining of hippocampus tissues. (**B**) Representative images of H&E staining of prefrontal cortex tissues. (**C**) Representative images of H&E staining of corpus striatum tissues. Scale bar: 50 μm. (**D**) Histological scores. All data are presented as the mean ± SEM. (*n* = 8; *P < 0.05, ***P < 0.001, ns = not significant).

In the NN group, hippocampal pyramidal neurons were uniformly arranged with clear demarcation, centrally located round nuclei, minimal chromatin, and prominent nucleoli, and no signs of inflammation. In the NH group, neuronal cells exhibited deeply stained nuclei with pyknosis, loss of cytoplasm-nucleus boundaries, and irregular shapes (black arrows), although inflammation was not evident. Similarly, in the HH group, neuronal cells showed deeply stained nuclei with pyknosis, indistinct cytoplasm-nucleus boundaries, and irregular morphology (black arrows), again without significant inflammation (Fig. 2A). In the NN group, neurons were abundant and regularly organized, with normal morphology, clear cytoplasm-nucleus boundaries, prominent nucleoli, and no detectable inflammation. In the NH group, no significant pathological alterations were observed. In the HH group, scattered neurons displayed nuclear pyknosis, boundary loss, and irregular shapes (black arrows), but no inflammation was noted (Fig. 2B). In both the NN and NH groups, neurons were abundant and orderly, with preserved morphology, clear cytoplasm-nucleus boundaries, and prominent nucleoli, and no signs of inflammation were detected. In the HH group, scattered neurons again showed nuclear pyknosis, loss of cytoplasm-nucleus boundaries, and irregular shapes (black arrows), although inflammation remained absent (Fig. 2C).

In summary, the NH and HH groups exhibited varying degrees of tissue injury across organs, with the HH group showing more pronounced damage to the lungs, liver, hepatic lobules, prefrontal cortex, and striatum compared with the NH group.

### Intestinal bacterial analysis of alpha and beta diversity

**Fig 3:**
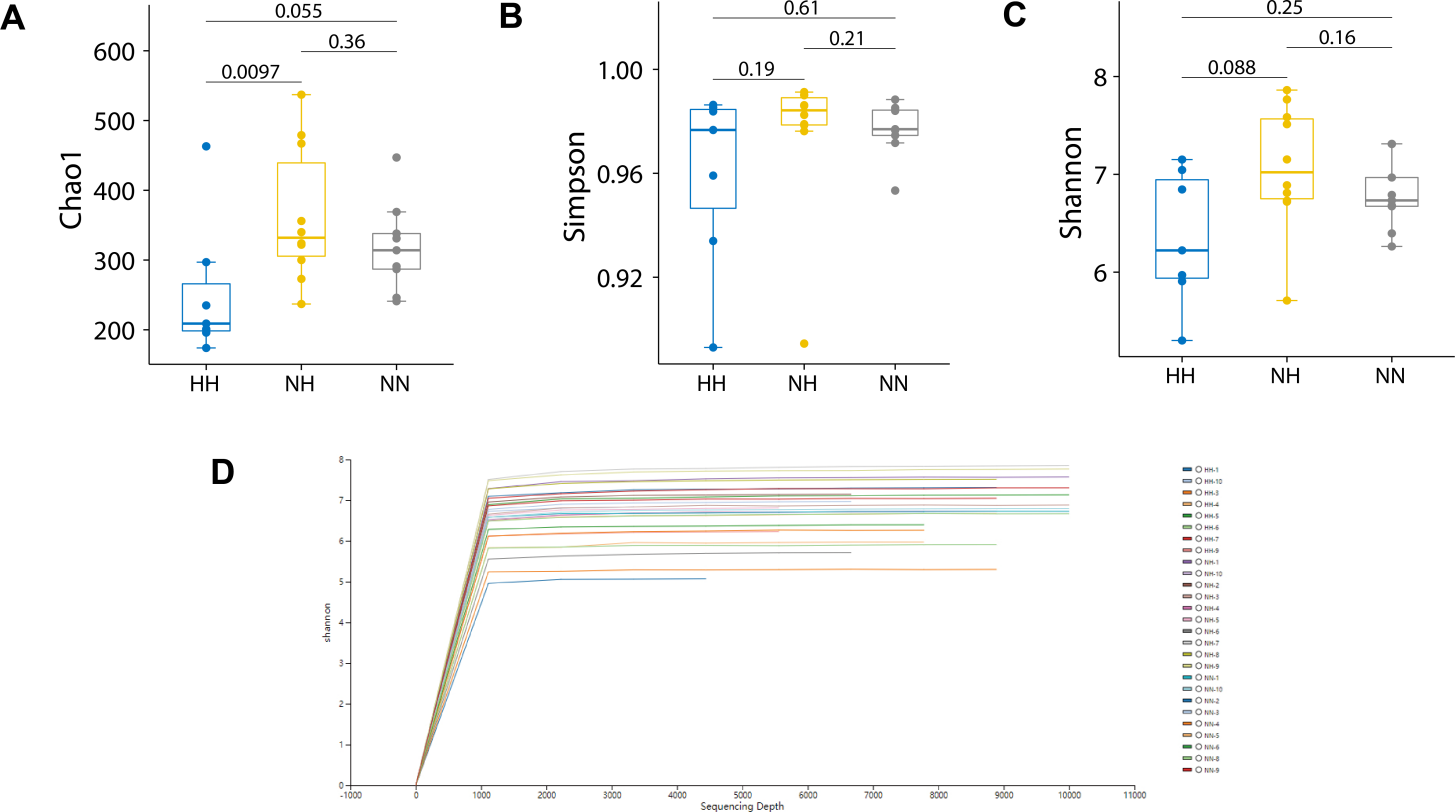
Intestinal bacterial alpha diversity. (**A**) Chao1 index, (**B**) Simpson’s index, and (**C and D**) Shannon dilution curve and index. HH refers to hypobaric hypoxia group, NH refers to normobaric hypoxia group, and NN refers to normobaric normoxia group.

The Chao1 index quantifies species abundance and richness by estimating the number of operational taxonomic units (OTUs) detected across all samples. The results indicated significant differences between the NH and HH groups, whereas neither differed significantly from the NN group (Fig. 3A). The Shannon and Simpson indices were used to assess species diversity, which reflects both species abundance and community evenness. At comparable levels of species abundance, higher evenness corresponds to greater overall diversity. An increase in the Shannon index and a decrease in the Simpson index similarly denote higher species diversity. Fig. 3B and C present the diversity index results. Owing to individual variation, neither the Simpson nor the Shannon indices differed significantly among the three groups. When sequencing depth reached 1,000, the Shannon rarefaction curve stabilized and no longer fluctuated with additional sequencing effort. Differences in curve height across samples reflected variation in microbial alpha diversity, effectively representing the diversity patterns among the three mouse gut communities (Fig. 3D).

**Fig 4:**
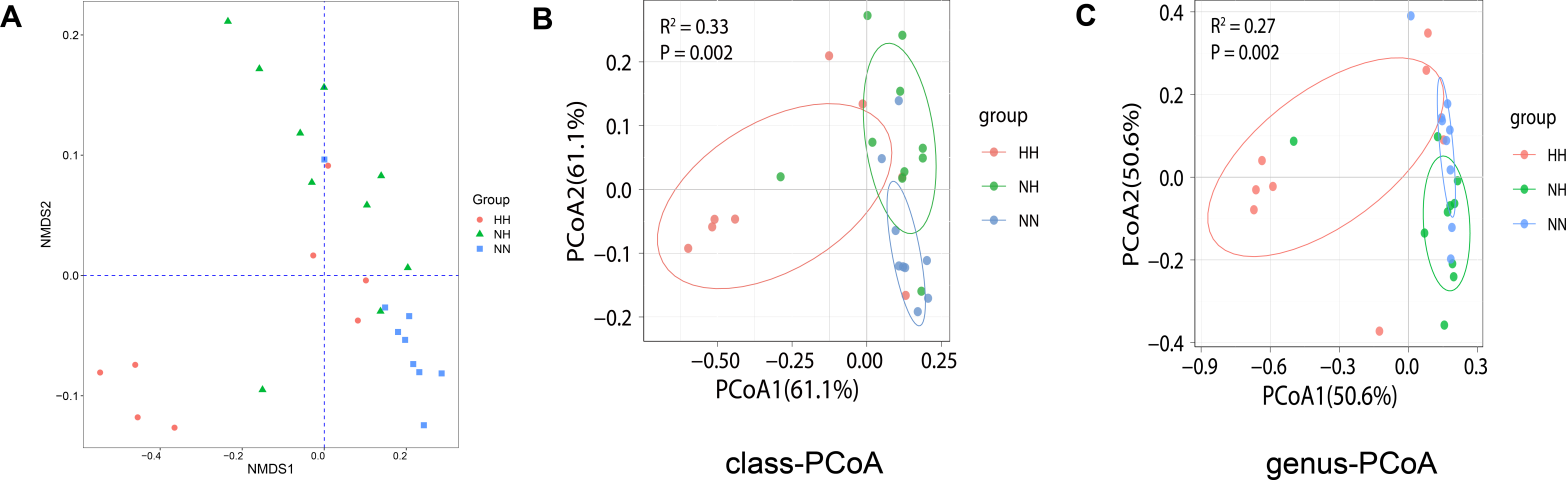
Beta diversity analysis compares the similarity of species diversity of different samples. (**A**) NMDS. (**B and C**) PCoA is divided into microbial class level and genus level. HH refers to hypobaric hypoxia group, NH refers to normobaric hypoxia group, and NN refers to normobaric normoxia group.

Beta diversity was used to compare and analyze differences in microbial community composition among samples. The analytical methods included non-metric multidimensional scaling (NMDS) and principal coordinate analysis (PCoA). The results showed higher similarity in the intestinal flora of mice within the same group and lower similarity across groups, indicating that HH markedly affected both the structure and abundance of the intestinal microbiota. NMDS analysis revealed significant differences among the three groups (Table S2), with the NN group exhibiting greater intra-group similarity than the NH and HH groups, which showed weaker repeatability (Fig. 4A). PCoA results further indicated clear group-level differences at the class level. The NN group displayed the strongest clustering, whereas the HH group exhibited the weakest clustering. PCoA at the genus level produced similar patterns (Fig. 4B and C).

### Analysis of the gut microbiota community structure

**Fig 5:**
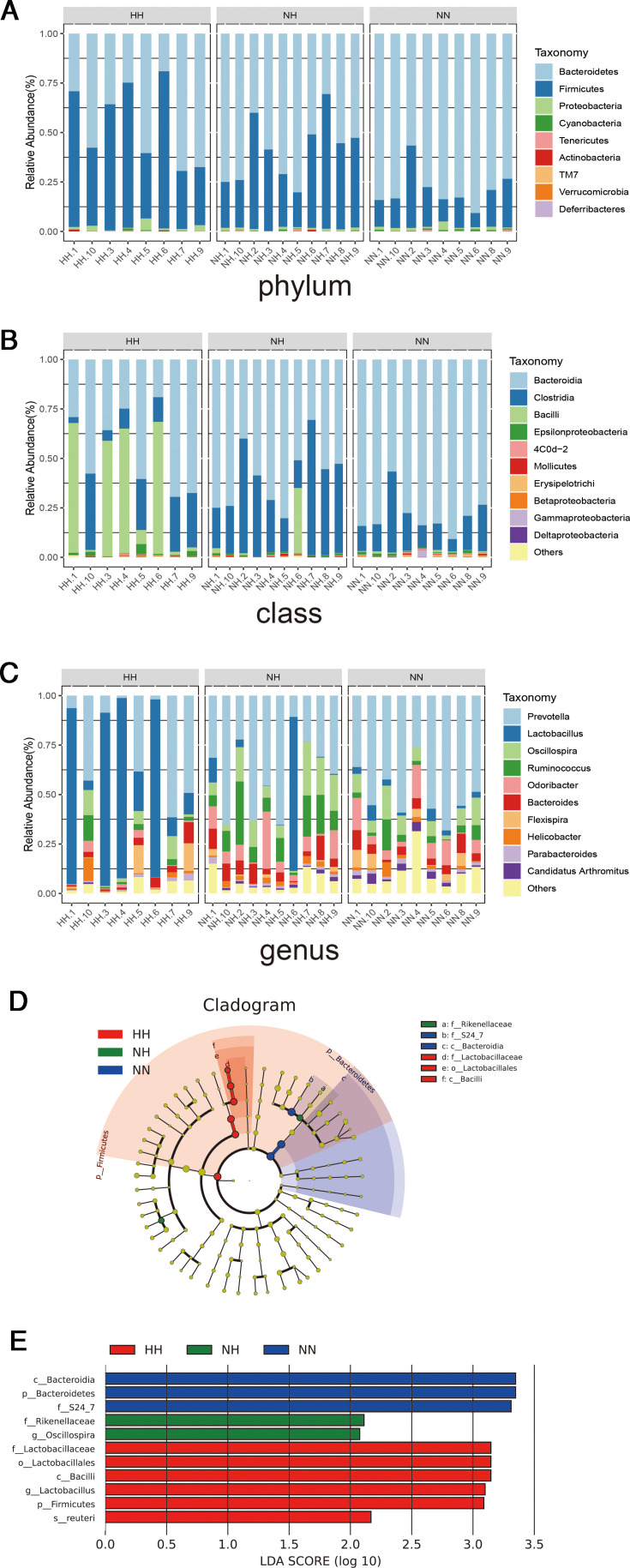
(**A–C**) Analysis of gut microbial community structure at phylum, class, and genus levels, with bars showing the species composition of each sample at different taxonomic levels. Color indicates species, and the length of the color block indicates the relative abundance of the species. Bacterial taxon differences between the HH and NH group feces microbiota by linear discriminant analysis effect size (LEfSe) in genus level. (**D**) LEfSe cladogram. (**E**) LEfSe histogram.

The plateau hypoxia environment altered the community structure and relative abundance of the intestinal microbiota (Fig. 5A). Species-level patterns in relative abundance were determined based on OTU annotations. The five dominant phyla were Bacteroidota, Bacillota, Proteobacteria, Cyanobacteria, and Mycoplasmatota. Compared with the NN and NH groups, the HH group showed an increased relative abundance of Proteobacteria.

As shown in Fig. 5B, the five most abundant classes were Bacteroidota, Clostridia, Bacilli, Campylobacterota, and 4C0d-2. Compared with the NN group and the NH group, the relative abundances of Bacilli and Campylobacterota differed in the HH group. At the genus level, Prevotella, *Lactobacillus, Oscillospira, Ruminococcus*, and *Odoribacter* were the top five taxa (Fig. 5C). The HH group showed marked shifts in *Lactobacillus*, while *Oscillospira* and *Ruminococcus* were most altered in the NH group. In the NN group, *Ruminococcus* and *Odoribacter* predominated. Overall, *Lactobacillus* and related genera differed substantially in relative abundance among the three groups.

The LEfSe identified several taxonomic branches with significant differences among the HH, NH, and NN groups (Table S3). To visualize the distribution of taxa across hierarchical levels, an evolutionary map from phylum to species was constructed. Bacteria within the class Bacilli were markedly more abundant in the HH group (Fig. 5D). Logarithmic discriminant analysis further indicated that *Lactobacillus* (Fig. S1) was the key genus-level biomarker in the HH group, whereas *Oscillospira* was enriched in the NH group (Fig. 5E).

### Functional prediction of gut microbiota

**Fig 6:**
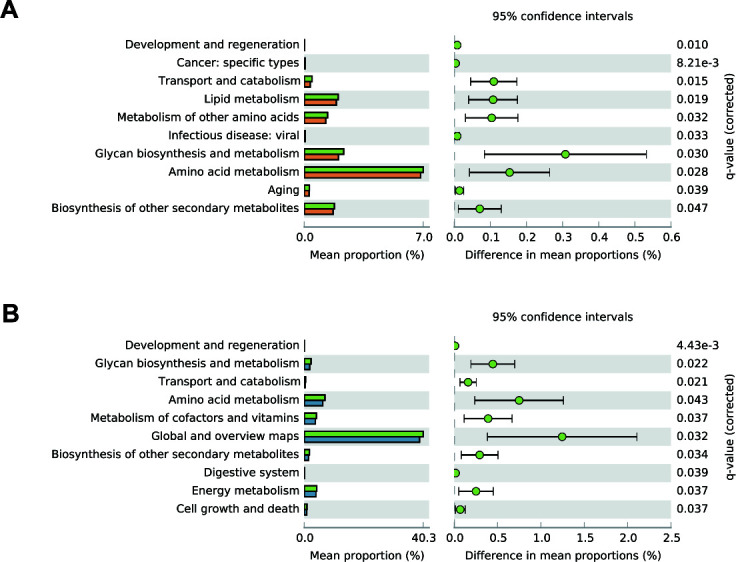
Functional analysis of the gut microbiota in each group of mice. (**A**) NH vs NN and (**B**) HH vs NN.

Because shifts in gut microbiota composition are often accompanied by functional changes, PICRUSt2 was used to predict gene functions associated with the enriched microbial communities. The three groups exhibited distinct functional profiles. In the NH group, pathways related to “Transport and catabolism,” “Lipid metabolism,” “Metabolism of other amino acids,” “Glycan biosynthesis and metabolism,” “Amino acid metabolism,” and “Biosynthesis of other secondary metabolites” were significantly enriched relative to the control group (Fig. 6A). In the HH group, “Glycan biosynthesis and metabolism,” “Amino acid metabolism,” “Metabolism of cofactors and vitamins,” “Biosynthesis of other secondary metabolites,” and “Energy metabolism” were significantly more enriched compared with the control group (Fig. 6B).

## DISCUSSION

Previous studies have shown that hypoxia can cause irreversible physiological damage (12, 13). Acute hypoxic responses induce pathological changes in multiple organs, including the heart, lungs, liver, and kidneys, and HH can markedly influence oxidative stress indices and organ-specific hypoxia sensitivity (24). The intestine is a key digestive organ whose unique vascularization and anatomy heighten its susceptibility to hypoxic stress (25). The gut microbiota also plays a central role in host health (26). Under normal conditions, it contributes to digestion and metabolism, nutrient absorption, immune regulation, and even behavioral modulation. However, disruption of this microbial balance poses a risk to host health (27).

In this study, we employed hypobaric and NH models to simulate equivalent P_I_O_2_ conditions. These two approaches produced distinct outcomes. Debate persists regarding the physiological differences between NH and HH. Millet et al. (28) attributed discrepancies to low-pressure effects, whereas Mounier and Brugniaux (29) argued that NH and HH elicit comparable physiological responses. Our histopathological analysis based on H&E staining demonstrated that, relative to the NN and NH groups, HH caused significantly greater organ injury in mice, particularly in the lungs, liver, hepatic lobules, prefrontal cortex, and striatum. In contrast, the severity of hippocampal damage was similar between the NH and HH groups compared to the control group. Gut microbiota sequencing further showed that both hypoxia models altered microbial community structure and relative abundance compared to the control group, indicating that these modeling approaches differentially affect the gut microbiota in mice.

Research indicates that high-altitude hypoxia exerts distinct effects on the digestive, cardiovascular, cerebrovascular, and nervous systems, leading to multiple forms of altitude sickness, including AMS, chronic polycythemia, cardiovascular disease, pulmonary edema, and cerebral edema (30). Histological analysis with H&E staining showed that, compared to the NN group, mice in both the NH and HH groups developed varying degrees of tissue injury in the lung, liver, hepatic lobules, prefrontal cortex, striatum, and hippocampus. Ibrahim et al. (31) reported that rats exposed to 2,100 m exhibited significant inflammatory cell infiltration relative to controls, presenting histopathological features closely resembling the pulmonary injury we observed in HH-exposed mice. Similarly, Chang et al. (32) documented significant neuropathological alterations in rats at 4,300 m, including cerebral neuronal edema, hippocampal neuronal degeneration, and pyknosis. These changes parallel the hippocampal abnormalities observed in both normobaric normoxic and hypobaric hypoxic mice. Their study also identified characteristic pulmonary and hepatic lesions at high altitude, including thickened alveolar walls, dilated and congested alveolar capillaries, alveolar edema, epithelial hyperplasia, neutrophil infiltration, central venous edema, disrupted hepatic cords, and inflammatory infiltration, all of which strongly align with pathological patterns in our HH group. Yuan et al. (33) further demonstrated that rapid ascent to plateau induces hepatic edema and inflammatory infiltration in rats, consistent with the liver injury we recorded in the HH group. A notable exploratory observation concerns the differential vulnerability of specific brain regions. Although hippocampal damage was pronounced and similar between the NH and HH groups, consistent with its known hypersensitivity to hypoxic stress (34), the striatum exhibited a distinct pattern. Striatal injury occurred exclusively in the HH group and was absent in the NH group. While the present findings offer primarily descriptive histological evidence, the mechanisms underlying these contrasting responses remain unclear. The distinct striatal susceptibility may relate to its central roles in motor regulation and neurotransmission (35), and previous studies indicate that HH conditions can markedly reduce striatal dendritic length (36–38). Future research integrating functional assessments, detailed morphological evaluation, and molecular analyses will be essential for clarifying these regional differences. The current results suggest that the striatum may represent a key target for investigating neurological impairment induced by HH.

The alpha diversity index reflects differences in bacterial groups within a sample. The results show no significant difference between the NN and HH groups, indicating consistent within-sample clustering. This suggests that simulating hypoxia by lowering the fraction of inhaled oxygen does not fully replicate the actual hypoxic environment. Beta diversity measures microbial compositional similarity between individuals. Significant differences were observed between the HH and NN groups, indicating that distinct hypoxic conditions altered intestinal microbial composition. The HH environment exerted a more pronounced effect on intestinal microbial aggregation. However, clustering bias may arise from genetics and other factors (39), including diet, cage effects, and sex.

Sequencing of the mouse gut microbiota showed that Bacteroidota was the dominant taxon at both the phylum and class levels. At the genus level, Prevotella was most abundant. The most notable changes in the HH group involved Bacillota, Bacillus, and *Lactobacillus*. Li and Zhao (40) reported that Han individuals living at high altitude were enriched in Bacillota, whereas those residing at low altitude exhibited greater enrichment of Bacteroidota. Jia et al. (41) further showed that the dominant gut bacteria in Tibetans from different regions of the Qinghai-Tibet Plateau included Bacteroidota, Bacillota, Proteobacteria, and Actinobacteria at the phylum level. The gut microbiotas of high-altitude mammals, including plateau pikas, Tibetans, Chinese Han populations living in Tibet, rhesus macaques, Tibetan antelopes, European mouflon, and blue sheep, exhibit a higher Firmicutes-to-Bacteroidetes (F/B) ratio than those of low-altitude mammals (14). Dong et al. further reported that Bacteroides is the dominant genus in low-altitude populations (42). Our findings align with these observations. Together, these consistent findings suggest that the HH condition may more closely replicate the native high-altitude environment and thereby induce comparable physiological responses, although this causal relationship requires further confirmation. Consistent with Dong et al.’s report of elevated *Lactobacillus* in high-altitude residents, our HH group showed a similar increase in lactobacilli relative to normobaric conditions. Xu et al. (43) found that *Lactobacillus* thrives under hypoxia, suggesting a potential adaptive advantage in the gut. Our results support this trend: *Lactobacillus* abundance increased under HH. Although previous studies have proposed beneficial roles for *Lactobacillus* in pathogen inhibition, immune regulation, antioxidant activity, and cholesterol metabolism (44), our study does not provide functional evidence to establish a causal protective effect. The observed increase may reflect a compensatory response to hypoxia-induced dysbiosis that helps maintain microbial homeostasis and limit intestinal injury. However, this interpretation remains speculative without mechanistic validation. Moreover, microbial responses to hypoxia vary across studies. For example, intermittent hypoxia reduced *Lactobacillus* populations in rats (45), indicating that altitude, modeling strategies, and exposure durations can produce divergent outcomes. Changes in *Ruminococcus* were more pronounced in the NH group. A previous study showed that *Ruminococcus* polysaccharides stimulate immune cells to produce inflammatory mediators such as TNF-α (46). In addition, active inflammatory bowel disease (IBD) is frequently associated with increased *Ruminococcus gnavus* (47). As a key component of the gastrointestinal immune barrier, the gut microbiota plays a central role in host health (48). Humans and their microbiota typically maintain a dynamic and mutually beneficial relationship, but environmental stressors such as hypoxia can disrupt this balance (49). Thus, hypoxia may impair gut barrier integrity in mice, increasing susceptibility to inflammatory bacterial expansion.

In the phylum-level analysis of intestinal microbiota, we observed that Proteobacteria showed an increasing trend in the HH group compared with the NN group. Although this difference was not statistically significant, the expansion of Proteobacteria suggests potential disruption of intestinal ecological balance or the onset of inflammatory responses. These findings indicate that Proteobacteria may contribute to the pathogenesis of hypoxia-related diseases or may themselves arise as a consequence of such conditions. Notably, Proteobacteria is considered the most unstable of the four dominant gut phyla (Bacillota, Bacteroidota, Proteobacteria, and Actinobacteria)(50). Its accumulation often reflects instability of the microbial community or a diseased host state (51), and rising Proteobacteria abundance is widely regarded as a diagnostic indicator of dysbiosis and disease risk (52–54). For instance, a study investigating gut microbiota composition in patients with colorectal adenoma (CRA) reported that Proteobacteria abundance increased from 66.0% to 77.4% in advanced CRA compared with normal mucosa (55).

Although dysbiosis can trigger numerous diseases, the mechanisms by which hypobaric and hypoxic conditions disturb gut microbiota and subsequently induce disease remain insufficiently understood. The emergence of distinct microbial communities is shaped by diverse factors, including host genetics, environment, delivery mode, diet, and pharmaceuticals (56). The disruption of oxygen gradients is a recognized driver of several intestinal disorders, such as IBD and colorectal cancer (57), both of which are frequently accompanied by microbiota imbalance. Therefore, hypoxia may alter microbial composition and structure, influencing metabolic pathways and contributing to functional impairments. Extensive research has underscored the essential role of the gut microbiota in maintaining host health. Probiotic supplementation, antibiotic therapy, and fecal microbiota transplantation (58) are three widely used strategies to modulate intestinal microbiota, each demonstrating efficacy in mitigating dysbiosis. Therefore, the effective restoration of disrupted microbial ecological balance represents a key therapeutic objective for microbiota-related diseases.

In this study, we compared the effects of NH and HH on the intestinal microbiota of mice. Our findings demonstrate that even under the same partial pressure of oxygen, the two hypoxia modes produced distinct impacts on microbial diversity, community structure, and key taxa. These differences warrant further investigation to clarify their mechanistic underpinnings. The gut microbiota remains extraordinarily complex, with numerous unknowns yet to be resolved. Moreover, understanding how hypoxia-induced microbial alterations influence host physiology represents an important step toward elucidating the broader interactions among host pathophysiological processes.

Although this study offers valuable insights, several limitations should be acknowledged. The sample size (*n* = 8 per group) was adequate to detect consistent phenotypic trends under controlled conditions, but larger cohorts are needed to strengthen statistical power and generalizability. The exclusive use of male mice, while intentional to avoid the estrous cycle-related variability and maintain hormonal homogeneity, restricts direct extrapolation to females. In addition, hypoxic exposure was simulated and did not fully replicate natural high-altitude conditions such as low temperature, increased solar radiation, or altered activity patterns. Furthermore, we did not assess behavioral or physiological parameters, and future studies should include these measurements to provide a more comprehensive perspective. Future validation in real high-altitude environments would therefore be beneficial. Other potential confounding factors also merit consideration. Although all mice received the same standard diet, minor differences in food consumption could have influenced microbiota composition and physiological responses. Coprophagy, a normal behavior in mice, may homogenize microbiota profiles within cages and either magnify or mask differences between groups. Standard housing conditions, including multiple mice per cage and randomized cage placement, were used to minimize cage effects, but their influence cannot be entirely excluded. Histological evaluations, although performed by trained investigators under blinded conditions, remain semi-quantitative. The 3-day acute exposure paradigm was suitable for capturing early responses, yet longer-term studies are essential for characterizing chronic adaptations. The inferred microbial functional profiles generated from 16S rRNA sequencing require confirmation using more direct approaches such as metagenomics or metabolomics. Finally, while the murine model provides a controlled experimental system, the interspecies differences in microbiota composition, immune function, and metabolism limit direct translation to humans. These constraints nevertheless highlight important directions for future research and opportunities to refine and extend the current findings.

## Data Availability

The raw PacBio 16S rRNA gene sequencing data have been deposited in the NCBI Sequence Read Archive (SRA) under BioProject accession number PRJNA1370043.

## SUPPLEMENTAL MATERIAL

File S1 spectrum.02214-25-s0001.pdf Updated SILVA taxonomy figures.

Supplementary data spectrum.02214-25-s0002.pdf Updated SILVA taxonomy tables.

Supplementary Figure S1 spectrum.02214-25-s0003.pdf New figure of LEfSe.

Table S1 spectrum.02214-25-s0004.xlsx Sequencing summary table.

Table S2 spectrum.02214-25-s0005.xlsx PERMANOVA/Adonis statistics with R², *P*-values.

Table S3 spectrum.02214-25-s0006.xlsx Complete list of significant taxa with LDA scores and FDR-adjusted *P*-values.

Table S4 spectrum.02214-25-s0007.xlsx Both alpha- and beta-diversity analyses (e.g., PCoA).

Table S5 spectrum.02214-25-s0008.xlsx All BioSample accession numbers (SAMN53415378–SAMN53415404, corresponding to NH, NN, and HH groups).

Table S6 spectrum.02214-25-s0009.xlsx Major discrepancies between the SILVA and Greengenes annotations.

ASM does not own the copyrights to Supplemental Material that may be linked to, or accessed through, an article. The authors have granted ASM a non-exclusive, world-wide license to publish the Supplemental Material files. Please contact the corresponding author directly for reuse.
