# Supplementary figures and images for: Comparative impacts of normobaric vs. hypobaric hypoxia on tissue integrity and gut microbiota in acute high-altitude murine models
Source: Microbiol Spectr. 2026 Feb 13;14(4):e02214-25. doi: 10.1128/spectrum.02214-25 (PMC13055316; doi:10.1128/spectrum.02214-25)

**Supplementary Figure S1** Linear Discriminant Analysis Effect Size (LEfSe) cladogram.

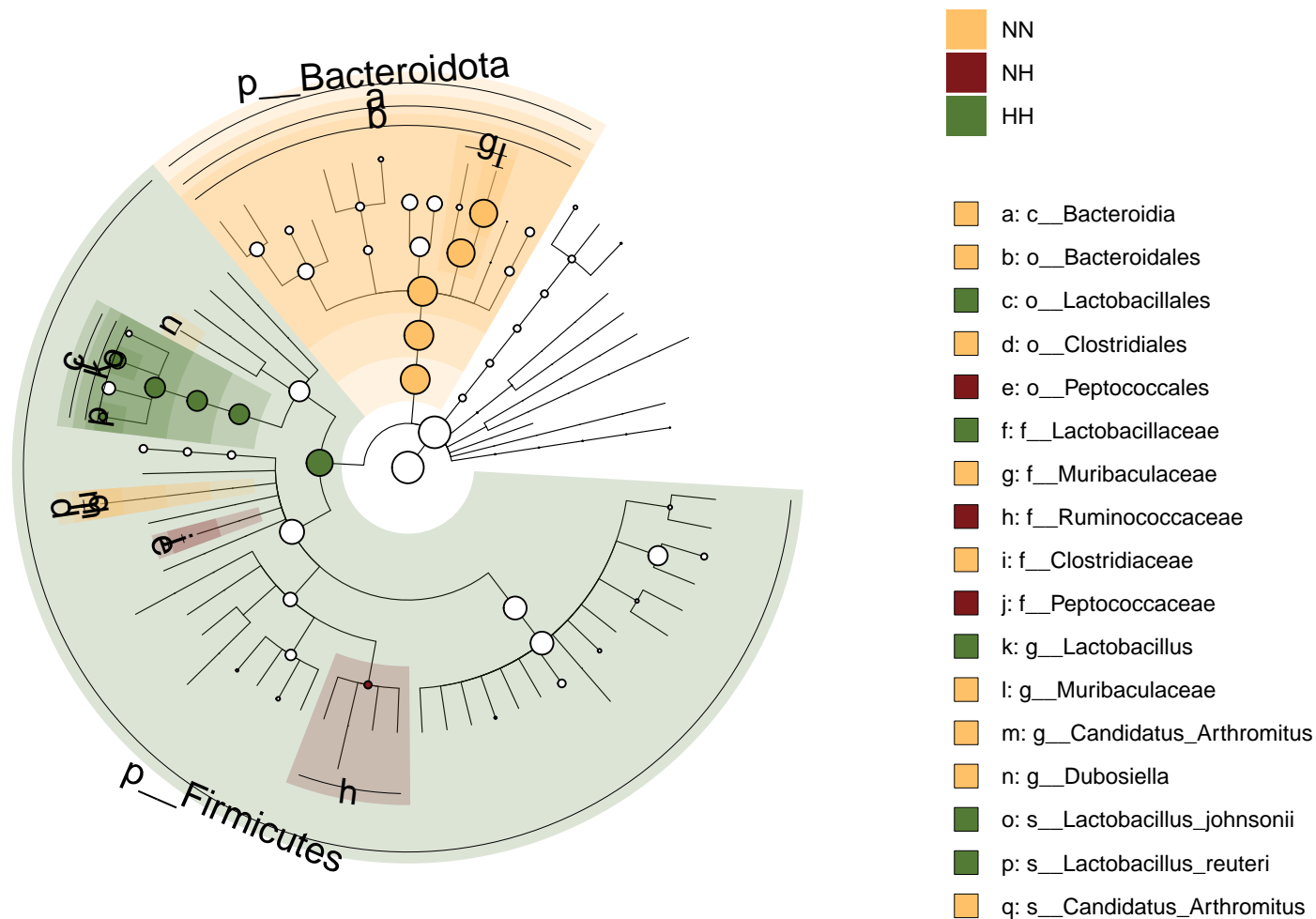

Supplement: Supplementary Figure S1 — New figure of LEfSe. [file spectrum.02214-25-s0003.pdf]
